# Millimeter Wave-Based Non-Destructive Biosensor System for Live Fish Monitoring

**DOI:** 10.3390/bios12070541

**Published:** 2022-07-20

**Authors:** Meng Wang, Yunyue Yang, Boyu Mu, Marina A. Nikitina, Xinqing Xiao

**Affiliations:** 1 Beijing Laboratory of Food Quality and Safety, College of Engineering, China Agricultural University, Beijing 100083, China; wangm.coe@cau.edu.cn (M.W.); yyy.coe@cau.edu.cn (Y.Y.); mby0527@cau.edu.cn (B.M.); 2 V.M. Gorbatov Federal Research Center for Foods Systems of RAS, 26, Talalikhina Street, 109316 Moscow, Russia; nikitinama@yandex.ru

**Keywords:** grouper, non-destructive, breathing rate, waterless transportation, millimeter wave radar

## Abstract

Waterless transportation for live grouper is a novel mode of transport that not only saves money, but also lowers wastewater pollution. Technical obstacles remain, however, in achieving intelligent monitoring and a greater survival rate. During live grouper waterless transportation, the stress response is a key indicator that affects the survival life-span of the grouper. Studies based on breathing rate analysis have demonstrated that among many stress response parameters, breathing rate is the most direct parameter to reflect the intensity. Conventional measurement methods, which set up sensors on the gills of groupers, interfere with the normal breathing of living aquatic products and are complex in system design. We designed a new breathing monitoring system based on a completely non-destructive approach. The system allows the real-time monitoring of living aquatic products’ breathing rate by simply placing the millimeter wave radar on the inner wall of the incubator and facing the gills. The system we developed can detect more parameters in the future, and can replace the existing system to simplify the study of stress responses.

## 1. Introduction

As a new and green transportation method, waterless live transportation can reduce the weight and volume of living aquatic products during transportation, improve transportation efficiency and density, save costs, and gradually become the development trend for live transportation [1,2]. Whether from an edible or ornamental point of view, live aquatic products tend to be more popular than chilled aquatic products, due to the influence of traditional concepts and life experiences [3,4]. The pearl gentian grouper is a hybrid of Epinephelus fuscoguttatus ♀ and male Epinephelus lanceolatus ♂ and has been successfully artificially bred, forming large-scale aquaculture in Hainan, China [5]. The pearl gentian grouper has become an important commercial and economic fish, due to its fast growth rate, strong disease resistance, fresh and tender meat, and rich nutrition, and demand for it is increasing [6]. In many low-temperature waterless live transportation experiments, we found that the pearl gentian grouper has a docile personality, is less stressed by environmental changes, and has strong vitality, which is very suitable as a species for this study.

**Figure 1:**
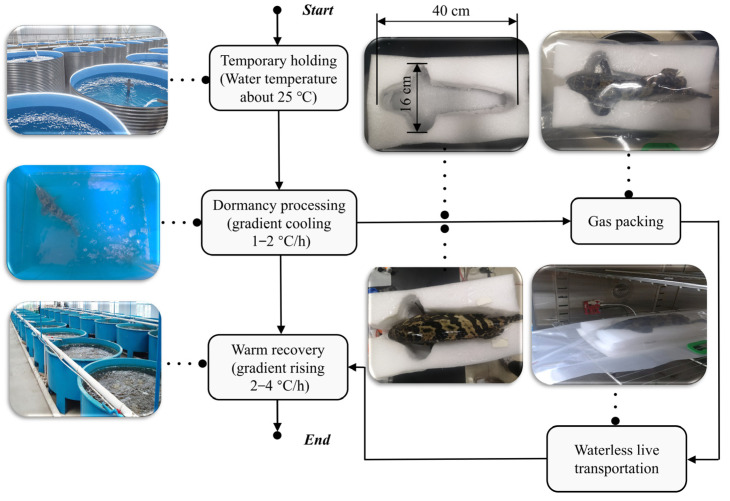
The whole procedure of the waterless transportation for live grouper.

Figure 1 shows the process of waterless transportation for live grouper. During waterless live transportation, living aquatic products are exposed to an inappropriate oxygen concentration for a long time, which renders them prone to stress reactions, resulting in tissue damage, decreased immunity, and even the death of the living aquatic products [7,8,9,10]. The stress parameters of the waterless live transportation of living aquatic products can be mainly divided into three categories: physiological stress performance; biochemical stress performance; and cumulative stress effects. However, the biochemical stress performance and cumulative stress effects mainly measure the energy consumption and survival quality of living aquatic products, and there is a certain hysteresis in the measurement and the measurement itself is destructive. The main indicators of the physiological stress response are respiratory rate, abnormal behavior, and gas parameters, such as O_2_ and CO_2_. The number of abnormal behaviors can intuitively reflect the severity of the stress, but it is still impossible to quantitatively calculate it. The change in gas concentration can reflect the state of respiratory stress, but the short-term fluctuation is small, and the sensor acquisition accuracy is very high. The respiratory rate can directly reflect a severe stress state, but the signal acquisition is difficult. When the living aquatic products are stressed, the breathing rhythm will be interrupted, resulting in abnormal breathing. When the living aquatic products are under pressure, the breathing rhythm will be interrupted, resulting in abnormal breathing. At this time, the movement of the gills should be checked to further determine the cause of the abnormality and take the next steps. Traditional methods of breathing rate detection are generally achieved by direct contact between the living aquatic product and wearable sensors or adhesive electrodes. However, wearable sensors can not only put pressure on the living aquatic product and limit its movement, but also create problems with fixation [11]. Therefore, working out how to realize the non-destructive measurement of breathing is an urgent problem. At present, the technical conditions for the intelligent detection of stress marker parameters of living aquatic products during live transportation are limited, and most of the stress signals collected by the physiological stress monitoring of living aquatic products are discrete signals [12]. Therefore, there is an urgent need to design an advanced biosensor device for the continuous monitoring of stress signals of the multi-category living aquatic products, to achieve effective monitoring and method regulation of the physiological stress of the living aquatic products in waterless live transportation. At the same time, with the advancement of automation and intelligent technology in all of the aspects of living aquatic product transportation, the monitoring of the respiration rate should progress to an intelligent dynamic visualization.

At present, the application of pulsed radar, Doppler radar, and frequency-modulated continuous-wave (FMCW) radar in the measurement of the respiration rate mainly focuses on the human body [13,14,15]. The FMCW radar, as opposed to pulse radar and Doppler radar, can detect the distance to target and Doppler information at the same time, and has become the standard in the field of breathing monitoring [16]. Higher frequency electromagnetic waves may also increase the phase modulation sensitivity in millimeter wave radar breath-detection devices [17].

This study mainly introduces a new non-destructive system for monitoring the respiration rate of living aquatic products during waterless live transportation, which can be used to directly reflect the stress behavior of living aquatic products, thereby enhancing their survival rate. The new system can remotely monitor the breathing rate, by using millimeter wave radar, and analyze the stress behavior changes of the living aquatic products through abnormal data. This system not only has the benefits of miniaturization, and low power consumption, but it may also be applied to a wider range of living aquatic products in the future, and it can detect many individuals at once. However, the fixed position of the sensor may not be determined under some conditions, resulting in the low robustness of the experimental data.

## 2. Material and Methods

### 2.1. Experimental Animals

A total of nine groupers were used in this experiment, four of which were pre-experimental. After being transported from Hainan to the Beijing seafood market, they were immediately transferred to laboratory aquariums for temporary breeding to ensure their activity. In the pre-experiment, four groupers were placed under the conditions of 4 °C, 7 °C, 10 °C, and 12 °C to find the suitable temperature for waterless live transportation. Four additional fish were also selected for validation trials at each of the four temperatures. Finally, the most suitable temperature was used for prolonged observation. The size of the grouper used in the final test was determined according to the previous multiple pre-experiments. The total length was 38 cm and the weight was about 1.25 kg. This experimental procedure followed the requirements for animal experiments.

### 2.2. Low-Temperature Waterless Live Grouper Transportation System

In the low-temperature waterless live transportation of living aquatic products, the grouper enters a dormant state, and the body’s metabolism and post-stress response are greatly reduced. Then, they will be in a relatively stable state [7]. The low-temperature waterless live transportation process of the grouper after reaching the laboratory can be divided into five main steps according to different operating characteristics:

**Step 1: Temporary holding.** Since the grouper belongs to tropical seawater fish, to make the groupers better adapt to the new environment, it is necessary to control the water temperature at 25 °C and add an appropriate amount of sea salt crystals to the aquarium. The fish are temporarily stored in the treated water for 24 h without feeding, and finally, the experiment is carried out according to the health of the grouper;

**Step 2: Dormancy.** Dormancy treatment plays a decisive role in the entire low-temperature waterless live transportation experiment, directly affecting the survival rate of the grouper. Through gradient cooling (1–2 °C/h), the grouper reaches the water temperature suitable for their dormancy, which is controlled at about 14°C in this experiment;

**Step 3: Gas packaging.** The groupers have just left the water at this stage; there will be many potential stress factors to stimulate, resulting in a strong stress response. Therefore, in the interests of ensuring that the fish have entered a low-temperature dormancy state, it is necessary to quickly and gently put the grouper into the packaging bags and aerate them with a certain amount of oxygen, carbon dioxide, etc., and promptly seal the bags. In addition, due to the shape of these fish, it is necessary to use specially made foam molds to keep the fish upright and avoid the squeezing of the gills, which affects the breathing monitoring effect;

**Step 4: Waterless live transportation.** This step is the core of the whole system. The different types of living aquatic products generally need to have different temperature parameters set for them. Since the grouper belong to tropical fish, they are different from the cold-water fish that the laboratory has executed before, and the temperature should generally be controlled at about 12 °C [2]. The temperature set in this study was 11–13 °C. The temperature of the incubator should be set up in advance, and after placing the living aquatic products in the incubator, the position of the sensor should be fixed immediately, and the door of the incubator closed, to avoid excessive temperature changes that may cause stress to the grouper and affect the experimental results;

**Step 5**: **Warming recovery.** When the transport process is over, the living aquatic products can be put back into the water; at this time the water temperature should be controlled at about 12 °C to avoid stress for the living aquatic products. It should be noted that the temperature change rate is about 2–4 °C/h. Finally, they need to be kept at room temperature for 1–2 days to restore vitality. It is worth noting that, to prevent the grouper from lacking oxygen in the water, the oxygen pump is used to provide oxygen to the grouper in steps one, two, and five.

### 2.3. Non-Destructive Breathing Rate Monitoring System (NDBRMS)

**Figure 2:**
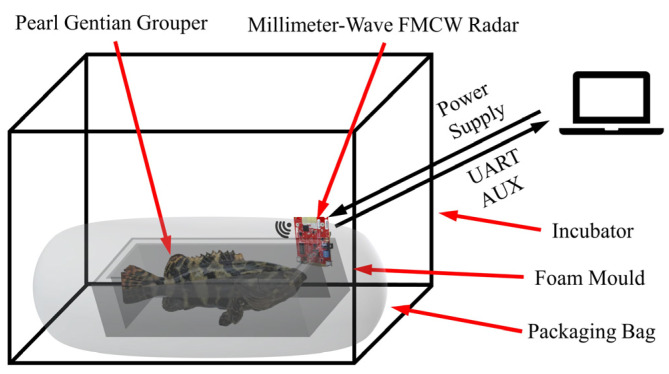
Schematic diagram of NDBRMS with the sensor fixed to the inner wall of the incubator and the radar antenna facing the gills.

The NDBRMS consists of the computer, the millimeter wave radar AWR1642 BOOST (Dallas, TX, USA), the waterless live transportation system, and the data processing program. The groupers that were in a low-temperature waterless live transportation environment are monitored by the NDBRMS. The schematic view of the NDBRMS is shown in Figure 2.

**Figure 3:**
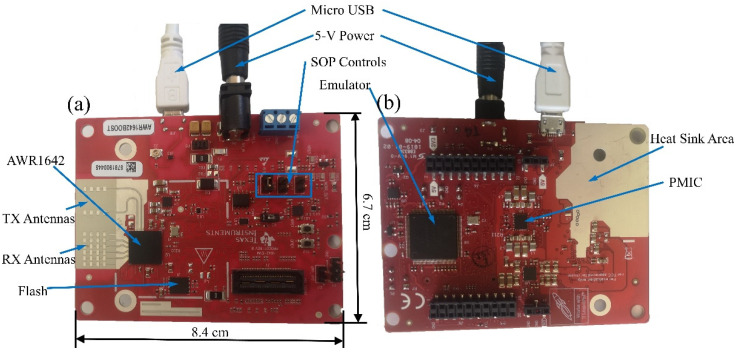
Prototype of the AWR1642 BOOST. (**a**) front view; (**b**) back view.

The AWR1642 BOOST used in this article consists of two Transmit (TX) Antennas, four Receive (RX) Antennas, AWR1642 Chip, Power Management IC (PMIC), Micro USB and 5-V Power Connector, Quad SPI (QSPI) flash drive, Heat Sink Area, and other components (Figure 3). The AWR1642 BOOST is an integrated single-chip frequency-modulated continuous wave (FMCW) radar sensor, operating at 77 GHz [18]. The FM pulse is alternately transmitted by the two TX Antennas. A single FM pulse has a period of 50 μs, a pulse interval of 7 μs, a transmitted sawtooth wave slope of 70 MHz/μs, and a frame rate of 50 ms [19]. The RX Antennas are responsible for receiving the reflected echo signal. The AWR1642 Chip is the core component in the AWR1642 BOOST, which integrates RF/analog system, radio processor subsystem, data processing system, and main control system. The PMIC is used to distribute the internal hardware voltages. The 5-V Power Connector is used for the radar board, to power the radar board. The flash drive is used to store programs and data. The host computer is connected to the radar board through the Micro USB on the onboard emulator, which can realize data programming and uploading. The Heat Sink Area reduces the radar temperature and ensures that it operates at the proper temperature. During the test, the radar antennas are facing the gills of the tested living aquatic products at a distance of about 40 cm, as shown in Figure 2. This minimizes outside interference and increases the signal strength associated with breathing. The size of the millimeter wave radar sensor is 8.4 × 6.7 cm. There are two cables connected to the computer in the experiment, namely, the Micro USB cable included in the AWR1642 package to communicate with the computer and the 5 V/2.5 A power cable to power the sensor. Data communication takes place through two ports, the Application/User UART (COMUART) and the Auxiliary Data Port (COMAUX). The basic tools for the AWR1642 measurement of the respiration rate are included in the millimeter wave automotive toolbox 2.9.1. The respiration rate of the groupers is calculated, using the procedures described in Section 2.4. Before the experiment, the movement of the grouper gills (Video S1, Supplementary Materials) was compared with the real-time curve of the TI’s GUI interface. When the change rule was basically the same, it was proved that the sensor was able to measure its respiration rate.

To evaluate the accuracy of the NDBRMS system in determining the heart rate, measurements were made using traditional camera techniques and manual counting methods. The camera was placed on the other side of the fish during the experiment and the respiration measurements were verified by manually observing the video after the test [20].

### 2.4. Data Processing

**Figure 4:**
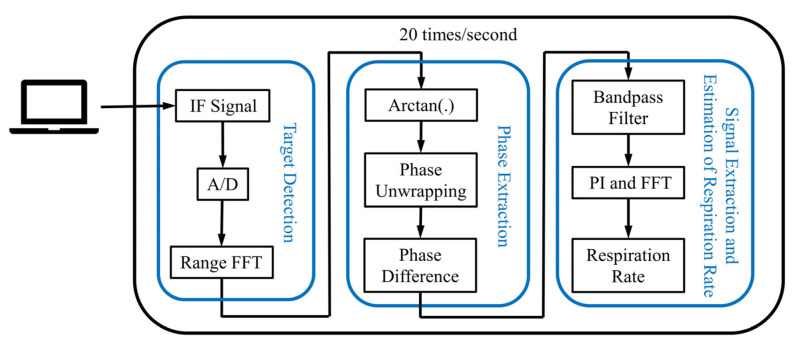
Data processing scheme. The sensor uses a frequency set to capture 20 times per second and is processed to determine the final breathing rate.

The breathing signal of the grouper is feeble, and the displacement caused by breathing is also millimeter-scale. The wavelength of the carrier wave at 77 GHz is 3.9 mm that resulted in a large modulation of the phase. The vibration displacement of the breathing signal may be determined using the phase change of the millimeter wave signal, thereby realizing the effective monitoring of the breathing rate [21,22]. However, disturbances, such as noise in the environment and the vibration of the incubator, are very serious and may drown out the breathing signal. Therefore, it is necessary to estimate the location of the fish first, and then process the phase signal from it, which can effectively reduce the interference of the external environment on the breathing signal. In Figure 4, the processing of the breathing signal is shown, which mainly includes four steps: target detection; phase extraction; signal extraction; and estimation of respiration rate.

#### 2.4.1. Target Detection

The millimeter wave radar transmits a FMCW signal *ST_t_* (Equation (1)) through the radio frequency module, receives the echo signal *SR_t_* (Equation (2)) reflected by the gills, mixes this with the transmitted signal and performs low-pass filtering, and then obtains the intermediate frequency (IF) signal *SIF_t_* (Equation (3)), through sampling and baseband signal processing.

(1) $$ST_{t}=AT\cdot \cos\left(2\pi f_{c}t+\pi \frac{B}{T_{c}}t^{2}+\phi_{t}\right)$$

(2) $$SR_{t}=AR\cdot \left[\cos\left(2\pi f_{c}\right)+\pi \frac{B}{T_{c}}{\left(t-t_{0}\right)}^{2}+\phi_{t-t_{0}}\right]$$

(3) $$SIF_{t}=AT\cdot AR\cdot \exp\left[j\left(2\pi f_{b}t+\psi_{t}\right)\right]$$

where *B* = 3.5 GHz is the bandwidth of the chirp; the range resolution is about 4 cm; the peak range bin is the 10th one; *f_c_* is the chirp-starting frequency; *AT* is the amplitude of the transmit signal; *AR* is the amplitude of the received signal; *T_c_* is the chirp duration; *ϕ_t_* is the phase noise from the transmitter; *t* is the time variable; *t*_0_ is the time delay generated at the radar receiver; *f_b_* is the beat frequency; and *ψ_t_* is the instantaneous phase of the IF signal. By performing the ADC and Range-FFT on the IF signal, the distance information between the grouper and the radar can be determined. The beam width is five degrees, which means that in actual measurement, it is about a 3.5 cm spot. Determining the optimal fish breathing signal extraction range-bin can be achieved by calculating the maximum average power in the range profile within the user-specified range limits, because the displacement motion will generate strong reflected signals and larger average power. The AW1642 mm wave radar can generate a virtual antenna array with one transmitter and eight receivers, and the clutter power can be better suppressed by superimposing eight channels of virtual IF signals. The respiration rate of the grouper in the low-temperature waterless live transportation should generally be 0.1–0.5 Hz, and the sampling frequency is set to 20 Hz to ensure no distortion or too much data.

#### 2.4.2. Phase Extraction

After the location of the grouper is determined, the complex range profile data in the range-bin can be extracted to achieve phase extraction. In this experiment, the arctangent demodulation method was used to recover the phase of the respiration signal (Equation (4)):

(4) $$\varphi_{t}=\arctan\left(I_{t}/Q_{t}\right)$$

where *I_t_* and *Q_t_* represent the baseband signals of I and Q channels, respectively; and *φ_t_* represents the phase value at time *t*. After the arctangent demodulation, the phase value is usually between [−π, π], because when the actual phase exceeds this range, phase wrapping occurs. The phase value is forced to add/subtract 2nπ (n is an integer), and then falls into the range, requiring it to be unwrapped to obtain the actual displacement curve. In practice, however, because the phases are continuous, we only need to execute phase unwrapping by adding or removing 2nπ from the phase (Equation (5)):

(5) $$p_{k}=\left\{\begin{array}{l} p_{k-1}-2\pi ,\varphi_{k}-\varphi_{k-1}>\pi \\ p_{k-1}+2\pi ,\varphi_{k-1}-\varphi_{k}>\pi \\ p_{k-1},\left|\varphi_{k}-\varphi_{k-1}\right|>\pi \end{array}\right.$$

where *k* ≥ 2, and is an integer; *p_k_* is the difference between the actual phase value and the measured value of the *k*th sampling point, which is an integer multiple of 2π, and the initial value is 0. The theoretical phase value after unwarping can be derived as shown in Equation (6):

(6) $${\varphi^{\prime }}_{t}=\varphi_{t}+p_{t}$$

where *φ′_t_* denotes the theoretical value of the phase, which is equal to the actual value. To enhance the respiration signal while suppressing the phase drift phenomenon, the final phase *φ″_t_* is determined by the method of adjacent consecutive phase differencing (Equation (7)):

(7) $${\varphi^{\prime \prime }}_{t}={\varphi^{\prime }}_{t}-{\varphi^{\prime }}_{t-1}$$

#### 2.4.3. Signal Extraction and Estimation of Respiration Rate

Since the breathing frequency is 0.1–0.5 Hz, the breathing signal can be further separated by a band-pass filter. In this study, a 4th-order IIR band-pass filter is designed, with the transfer function *H*(*z*) shown in Equation (8):

(8) $$H\left(z\right)=\prod_{k=1}^{N}\frac{b_{0}^{k}+b_{1}^{k}z^{-1}+b_{2}^{k}z^{-2}+b_{3}^{k}z^{-3}+b_{4}^{k}z^{-4}}{1+a_{1}^{k}z^{-1}+a_{2}^{k}z^{-2}+a_{3}^{k}z^{-3}+a_{4}^{k}z^{-4}}$$

where *k* is the number of cascade levels; *N* is the number of delay cells; *b* is the positive term coefficient; and *a* is the inverse coefficient. Using the “findpeaks” function in MATLAB R2019a (version 9.6 Release March 2019, The Mathworks, Natick, Boston, MA, USA), the detection of the local maxima was implemented and used to estimate the respiration rate of the grouper. The average breathing rate *R* is calculated by the number of peaks *n_r_* (within one-minute interval) and the time *T* between the first and last peak (Equation (9)). On the other hand, by using FFT to convert the signal to the frequency domain, find the frequency at the peak, and compare it with the calculation result of the peak interval (PI) method to determine the final result:

(9) $$R=\left(n_{r}-1\right)/T$$

## 3. Results and Discussion

**Figure 5:**
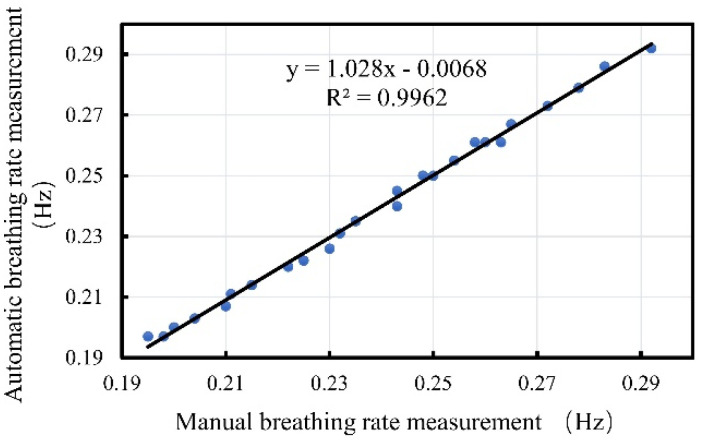
Comparison of the effects of manual observation and the NDBRMS system for measuring breathing rate.

Figure 5 shows a comparison of the effects of manual observation and the NDBRMS system for measuring the respiration rates. The performance of the NDBRMS system was verified by recording a video. A total of 25 measurements were recorded for four groupers, and it can be seen that the NDBRMS results are very close to the true respiration rate.

**Figure 6:**
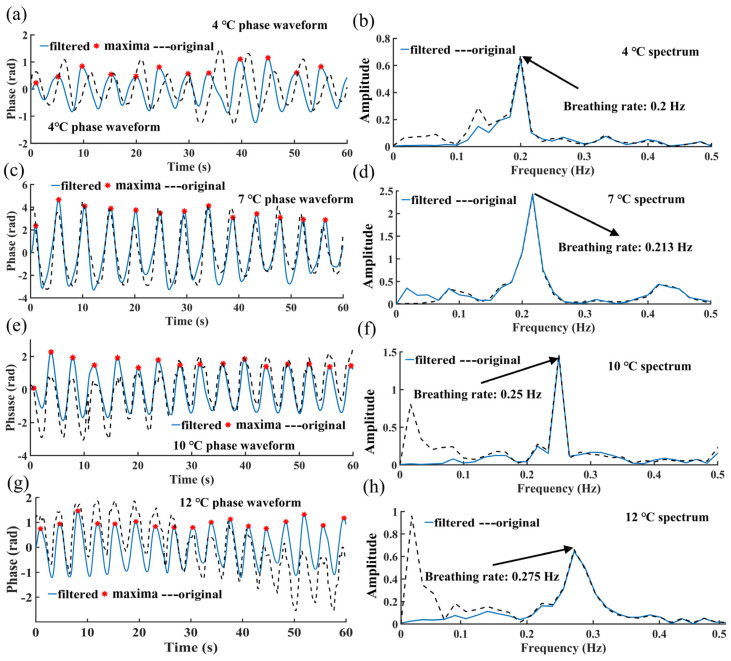
Breathing signal filtering and PI in preliminary experiments. (**a**) Breathing signal phase waveform at 4 °C; (**b**) Breathing signal spectrum at 4 °C; (**c**) Breathing signal phase waveform at 7 °C; (**d**) Breathing signal spectrum at 7 °C; (**e**) Breathing signal phase waveform at 10 °C; (**f**) Breathing signal spectrum at 10 °C; (**g**) Breathing signal phase waveform at 12 °C; (**h**) Breathing signal spectrum at 12 °C. The blue solid line represents the filtered, the black dashed line represents the measured original and the red stars represent the local maximum used to calculate the respiration rate.

Figure 6 shows the results of the preliminary experiment using NDBRMS to detect the respiration rate of the grouper. This not only shows the feasibility of the experiment, but also provides a temperature suitable for the grouper for waterless live transportation. From the overall picture in Figure 6, we can see that the respiration rate increases significantly with temperature. At 4 °C, the respiration rate of the living aquatic products is about 0.2 Hz, as shown in Figure 6a,b. Figure 6c,d show that at 5 °C, the living aquatic products have a respiration rate of roughly 0.213 Hz. Figure 6e,f demonstrate the respiration rate of the living aquatic products at 10 °C. Figure 6g,h reveal that the respiration rate of the living aquatic products is around 0.275 Hz at 12 °C. The PI and FFT calculations of the breathing frequency results are within 0.05 Hz of each other. Again, we can find that the use of filtering is necessary, as it makes the calculation results more accurate. In the phase waveform, the filtered waveform can calculate an accurate respiration rate using PI. In the spectrum, the filtered waveform highlights the actual fish respiration rate PPT values. Although the respiration results show that at 4–10 °C, the respiration rate of the grouper is lower and the metabolism is smaller, the survival time of the fish at this temperature is greatly reduced, the vitality is extremely low, and there is no stress response. At 12 °C, the grouper can be transported without water for a longer time, with better vitality, and a higher recovery success rate, so the final experiment determined the use temperature to be 12 °C. It is worth noting that in the pre-experiment, we tried to compare existing acceleration sensors with the system we developed, but unfortunately, due to the mucus and respiratory properties of the grouper body surface, (Video S1, Supplementary Materials), it was not possible to measure it, even with a sensor the size of a fingernail.

**Figure 7:**
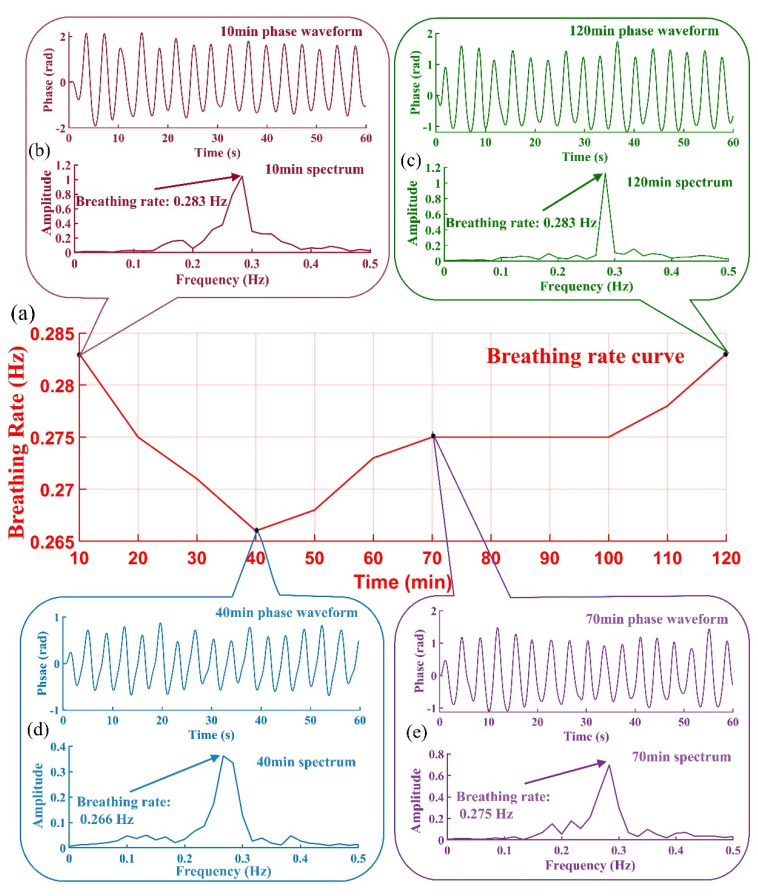
(**a**) Two-hour ‘normal’ breathing rate change curve; (**b**) Breathing signal phase waveform and its spectrum at 10 min; (**c**) Breathing signal phase waveform and its spectrum at 120 min; (**d**) Breathing signal phase waveform and its spectrum at 40 min; (**e**) Breathing signal phase waveform and its spectrum at 70 min.

Figure 7a shows the two-hour respiration rate curve monitored by NDBRMS in the 12 °C waterless live transportation experiment. It is worth mentioning that the use of phase changes over time to describe the fish breathing depth (the size of the gill opening) seems to be worthy of further discussion. If the movement of the gills is directly in the direction of the radar beam, the phase transition can be used to calculate the displacement. Since the motion may not be along that direction, it will involve the dot product between the gill surface and the radar beam motion. This phase information provides an upper limit to the displacement of the gills, since the phase change depends on only the motion along the direction of the beam and not perpendicular to it. From Figure 7b–e, within a short period, the breathing depth of the grouper is not uniform, and deep breathing seems to occur at intervals. From the overall respiration rate change in 2 h, the respiration rate change is relatively stable, and the respiration rate change is within 0.02 Hz, so in practical applications, if the respiration rate changes greatly, it can be considered that the grouper has a stress response, but the relationship between the change of fish respiration depth and fish stress response still needs to be further studied. Feng et al. found similar results to this paper in the waterless live transportation experiment of salmon. Within two hours, the respiration of the grouper did not undergo severe mutation when it was stressed [11]. Fan et al. and Das et al. also found that the respiration rate of the grouper increased with the temperature, and the respiration rate was about 0.27 Hz at 12 °C [23,24]. When the temperature drops to 12 °C, the respiration rate changes very slowly with the temperature drop, and the survival time of the fish below this temperature will be seriously affected [25].

The main advantage of the new system compared to the existing fish respiration monitoring system under waterless conditions is that it does not require the installation of sensors in the gills of the grouper, is essentially harmless, and does not affect the whole process of fish respiration, thus allowing for measurements to be taken under more natural conditions. The fixation of the sensor can cause problems in practical applications, as the fish can be subjected to strong stress reactions during transportation due to vibration or temperature changes, etc., causing the fish to move significantly, making it impossible for the sensor to be aimed squarely at the gills of the fish and to measure the accurate fish respiration rate. If the grouper moves sideways, the gills can move out of the radar beam spot size, making the signal too weak to detect. If the gills are turned too far at an angle as they move, the radar beam may not return to the radar and the entire signal may be lost. The non-destructive method is based on the detection of gill movement during waterless live transportation by frequency-modulated continuous-wave (FMCW), which further responds to the respiration rate of the fish. Due to the sensor itself, strong signal interference in the vicinity should be avoided, such as the change of chest phase caused by human breathing. During data processing, signal interference can be effectively eliminated by filtering, as described in Section 2, but this requires that we know the range of respiration rate of the fish under certain conditions.

Since the grouper selected for the experiment could not be placed vertically in the bag, a suitable foam mold was chosen to prevent the gills from being squeezed, which could lead to inaccurate measurement data. However, in practical large-scale applications, the foam molds may make the whole waterless live transportation process cumbersome, so it is also important to find a more suitable fixation method for the grouper. It is also the existence of the foam molds that makes the non-destructive measurement methods, such as laser ranging, hard. According to the Nyquist–Shannon sampling theorem, the system can theoretically measure a maximum respiration rate of 10 Hz, while in practice, due to the presence of noise, the maximum respiration rate is 2.5 Hz, which is one-fourth of the theoretical value. The respiration rate of the different species of fish during the waterless live transportation is generally less than 1 Hz [4,11]. Setting a higher sampling frequency can increase the maximum respiration rate, and even make it possible to monitor the heart rate of grouper in the future.

The NDBRMS can not only monitor the breathing rate changes of one grouper but also detect the breathing rate changes of multiple groupers, as studies have been successfully conducted to achieve the simultaneous detection of multiple individual vital signs [19,26]. If the other gill is located in other range-bins, it can also be separated by the algorithm, but it is more difficult. Considering the cost of sensors and signal interference, multiple radar sensors should not be used in the same scene, but one sensor should be used to monitor multiple fish, to reflect the other stress responses in the entire environment. When the biosensor is attached to the packaging bag, the hardness of the packaging bag should be guaranteed to prevent the phase change caused by the deformation of the packaging bag. Millimeter waves can penetrate any insulating material, metal materials cannot penetrate at all, and wood, paper, and glass have moderate penetration ability. One centimeter of plastic material attenuates about 0.8 dB, and one centimeter of wood attenuates about 5 dB. When this system is applied to shellfish, such as shrimps and crabs, the weakening of the signal by the shell needs to be overcome. For other large or small fish, a gill movement that is too large or too small may be beyond detection. In reality, the real-time monitoring of fish can be achieved directly using the GUI interface introduced by TI, whose internal algorithms require only a few parameter changes.

## 4. Conclusions

The main objective of this study was to develop a non-destructive system for respiration-rate detection in groupers, to simplify the process of stress response monitoring of living aquatic products during waterless live transportation. The feasibility of the NDBRMS was fully validated through pre-experiments and two hours of continuous monitoring. The NDBRMS can accurately measure the respiration rate of the grouper by simply fixing the sensor inside the incubator without attaching anything to the grouper. Although other limitations may be encountered in practice, this study presents a non-destructive measurement method that can be initially implemented during waterless live transportation. The fact that different species of fish have similar physiological characteristics also ensures the applicability of the system to other species. In addition, the respiration rate of some other marine organisms, such as lobsters and crabs, can also be measured, using the principles of this system. Moreover, non-destructive measurement of the heart rates of living aquatic products during waterless live transportation may be possible in the future.

## Data Availability

Not applicable.

## Supplementary Materials

The following supporting information can be downloaded at: https://www.mdpi.com/article/10.3390/bios12070541/s1, Video S1: 15 s breathing video.

## References

[1] Feng H., Zhang M., Gecevska V., Chen B., Saeed R., Zhang X. (2022). Modeling and evaluation of quality monitoring based on wireless sensor and blockchain technology for live fish waterless transportation. Comput. Electron. Agric..

[2] Wang W., Xu J., Zhang W., Glamuzina B., Zhang X. (2021). Optimization and validation of the knowledge-based traceability system for quality control in fish waterless live transportation. Food Control.

[3] Zhang Y., Wang W., Yan L., Glamuzina B., Zhang X. (2019). Development and evaluation of an intelligent traceability system for waterless live fish transportation. Food Control.

[4] Wang W., Zhang Y., Liu Y., Adányi N., Zhang X. (2020). Effects of waterless live transportation on survivability, physiological responses and flesh quality in Chinese farmed sturgeon (*Acipenser schrenckii*). Aquaculture.

[5] Zhang Z., Yang Z., Ding N., Xiong W., Zheng G., Lin Q., Zhang G. (2018). Effects of temperature on the survival, feeding, and growth of pearl gentian grouper (female *Epinephelus fuscoguttatus* × male *Epinephelus lanceolatus*). Fish. Sci..

[6] Zhang W., Tan B., Ye G., Wang J., Dong X., Yang Q., Chi S., Liu H., Zhang S., Zhang H. (2019). Identification of potential biomarkers for soybean meal-induced enteritis in juvenile pearl gentian grouper, *Epinephelus lanceolatus*♂ × *Epinephelus fuscoguttatus* ♀. Aquaculture.

[7] Xu Z., Regenstein J.M., Xie D., Lu W., Ren X., Yuan J., Mao L. (2018). The oxidative stress and antioxidant responses of Litopenaeus vannamei to low temperature and air exposure. Fish Shellfish Immunol..

[8] Zeng P., Chen T., Shen J. (2014). Effects of cold acclimation and storage temperature on crucian carp (*Carassius auratus gibelio*) in a waterless preservation. Fish Physiol. Biochem..

[9] Lorite G.S., Selkälä T., Sipola T., Palenzuela J., Jubete E., Viñuales A., Cabañero G., Grande H.J., Tuominen J., Uusitalo S. (2017). Novel, smart and RFID assisted critical temperature indicator for supply chain monitoring. J. Food Eng..

[10] Fan X., Qin X., Zhang C., Zhu Q., Chen J., Chen P. (2019). Metabolic and anti-oxidative stress responses to low temperatures during the waterless preservation of the hybrid grouper (*Epinephelus fuscogutatus*♀ × *Epinephelus lanceolatus*♂). Aquaculture.

[11] Feng H., Zhang M., Zhang L., Chen B., Zhang X. (2021). Evaluation of dynamic stress level and physiological change for live salmon in waterless and low-temperature transportation. Aquaculture.

[12] Nie X., Zhang F., Wang T., Zheng X., Li Y., Huang B., Zhang C. (2019). Physiological and morphological changes in Turbot (*Psetta maxima*)gill tissue during waterless storage. Aquaculture.

[13] Vandersmissen B., Knudde N., Jalalvand A., Couckuyt I., Bourdoux A., De Neve W., Dhaene T. (2018). Indoor Person Identification Using a Low-Power FMCW Radar. IEEE Trans. Geosci. Remote Sens..

[14] Wang Y., Ren A., Zhou M., Wang W., Yang X. (2020). A novel detection and recognition method for continuous hand gesture using fmcw radar. IEEE Access.

[15] Piotrowsky L., Jaeschke T., Kueppers S., Siska J., Pohl N. (2019). Enabling High Accuracy Distance Measurements with FMCW Radar Sensors. IEEE Trans. Microw. Theory Tech..

[16] Mercuri M., Sacco G., Hornung R., Zhang P., Visser H.J., Hijdra M., Liu Y.H., Pisa S., Van Liempd B., Torfs T. (2021). 2-D Localization, Angular Separation and Vital Signs Monitoring Using a SISO FMCW Radar for Smart Long-Term Health Monitoring Environments. IEEE Internet Things J..

[17] Chuang H.R., Kuo H.C., Lin F.L., Huang T.H., Kuo C.S., Ou Y.W. (2012). 60-GHz millimeter-wave life detection system (MLDS) for noncontact human vital-signal monitoring. IEEE Sens. J..

[18] Chahrour H., Dansereau R.M., Rajan S., Balaji B. (2021). Target Detection through Riemannian Geometric Approach with Application to Drone Detection. IEEE Access.

[19] Singh A., Rehman S.U., Yongchareon S., Chong P.H.J. (2021). Multi-Resident Non-Contact Vital Sign Monitoring Using Radar: A Review. IEEE Sens. J..

[20] Tuan S.A., Rustia D.J.A., Hsu J.T., Lin T. (2022). Te Frequency modulated continuous wave radar-based system for monitoring dairy cow respiration rate. Comput. Electron. Agric..

[21] Cen S.H., Newman P. Precise Ego-Motion Estimation with Millimeter-Wave Radar under Diverse and Challenging Conditions. Proceedings of the 2018 IEEE International Conference on Robotics and Automation, ICRA 2018.

[22] Zhao P., Lu C.X., Wang J., Chen C., Wang W., Trigoni N., Markham A. MID: Tracking and identifying people with millimeter wave radar. Proceedings of the 2019 15th International Conference on Distributed Computing in Sensor Systems (DCOSS).

[23] Fan X., Qin X., Zhang C., Chen J., Zhu Q. (2018). Effects of temperature on metabolism function and muscle quality of grouper during process of keeping alive with water. Trans. Chinese Soc. Agric. Eng..

[24] Das S.K., De M., Ghaffar M.A., Noor N.M., Mazumder S.K., Bakar Y. (2021). Effects of temperature on the oxygen consumption rate and gill fine structure of hybrid grouper, *Epinephelus fuscoguttatus* ♀ × *E. Lanceolatus* ♂. J. King Saud Univ.-Sci..

[25] Estudillo C.B., Duray M.N. (2003). Transport of hatchery-reared and wild grouper larvae, *Epinephelus* sp.. Aquaculture.

[26] Ahmad A., Roh J.C., Wang D., Dubey A. Vital signs monitoring of multiple people using a FMCW millimeter-wave sensor. Proceedings of the 2018 IEEE Radar Conference (RadarConf18).

