# Supplementary figures and images for: Millimeter Wave-Based Non-Destructive Biosensor System for Live Fish Monitoring
Source: Biosensors (Basel). 2022 Jul 20;12(7):541. doi: 10.3390/bios12070541 (PMC9313324; doi:10.3390/bios12070541)

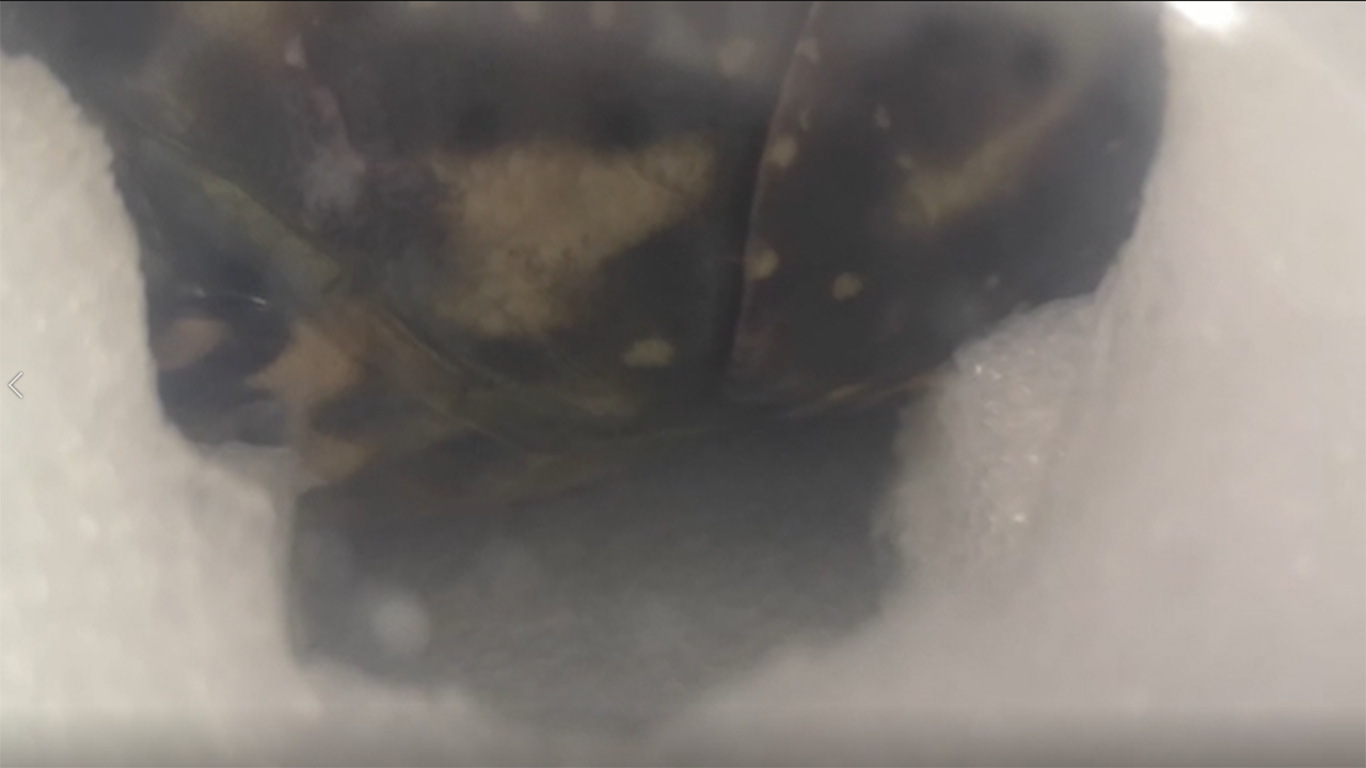

Supplement: Supplementary file 1 [file biosensors-12-00541-s001.zip › Video Cover.tif]
